# Black blow fly (Diptera: Calliphoridae) bacterial symbionts inform oviposition site selection by stable flies (Diptera: Muscidae)

**DOI:** 10.1093/jisesa/ieae040

**Published:** 2024-04-10

**Authors:** Sophie Hennig, Emmanuel Hung, Claire Gooding, Gerhard Gries

**Affiliations:** Department of Biological Sciences, Simon Fraser University, Burnaby, BC V5A 1S6, Canada; Department of Biological Sciences, Simon Fraser University, Burnaby, BC V5A 1S6, Canada; Department of Biological Sciences, Simon Fraser University, Burnaby, BC V5A 1S6, Canada; Department of Biological Sciences, Simon Fraser University, Burnaby, BC V5A 1S6, Canada

**Keywords:** Diptera, Muscidae, Calliphoridae, semiochemical, larval habitat

## Abstract

Larval habitats of blood-feeding stable flies, *Stomoxys calcitrans* (L.) (Diptera: Muscidae), overlap with foraging sites of black blow flies, *Phormia regina* (Meigen) (Diptera: Calliphoridae). We tested the hypothesis that bacteria in blow fly excreta inform oviposition decisions by female stable flies. In laboratory 2-choice bioassays, we offered gravid female stable flies fabric-covered agar plates as oviposition sites that were kept sterile or inoculated with either a blend of 7 bacterial strains isolated from blow fly excreta (7-isolate-blend) or individual bacterial isolates from that blend. The 7-isolate-blend deterred oviposition by female stable flies, as did either of 2 strains of *Morganella morganii* subsp. *sibonii*. Conversely, *Exiguobacterium* sp. and *Serratia marcescens* each prompted oviposition by flies. The flies’ oviposition decisions appear to be guided by bacteria-derived semiochemicals as the bacteria could not be physically accessed. Oviposition deterrence caused by semiochemicals of the 7-isolate-blend may help stable flies avoid competition with blow flies. The semiochemicals of bioactive bacterial strains could be developed as trap lures to attract and capture flies and deter their oviposition in select larval habitats.

## Introduction

The stable fly, *Stomoxys calcitrans* (L.) (Diptera: Muscidae), is a cosmopolitan blood-feeding pest of livestock (Rochon et al. 2021). Stable fly larvae develop in ephemeral sites of decaying organic matter, such as crop residues or livestock feed mixed with animal fecal and urinary excretions (Williams et al. 1980, Meyer and Petersen 1983, Cook et al. 2018). The removal or amendment of such larval development sites is an effective tactic of stable fly management (Thomas et al. 1996, Cook 2020).

To locate larval development sites, gravid female stable flies exploit odors and gases emanating from these sites (Baleba et al. 2019, Nayani et al. 2023a). Certain species of bacteria not only produce semiochemicals that attract gravid female flies and elicit oviposition but are also essential for larval development (Lysyk et al. 1999, Romero et al. 2006, Albuquerque and Zurek 2014).

During feeding, defecation, and oviposition, flies inoculate sites with bacterial symbionts that are present in their regurgitate and feces or on their eggs (Lam et al. 2007, 2009, Zheng et al. 2013, Holl and Gries 2018, Uriel et al. 2020). Deposited symbionts then produce volatile semiochemicals which, in turn, recruit more flies to a resource site (Uriel et al. 2020). This “fly factor” phenomenon was first observed in foraging house flies, *Musca domestica* (L.) (Diptera: Muscidae) (Barnhart and Chadwick 1953), and also mediates aggregated oviposition and/or feeding in black blow flies, *Phormia regina* (Meigen) (Diptera: Calliphoridae), green bottle flies, *Lucilia sericata* (Meigen) (Diptera: Calliphoridae), and face flies, *Musca autumnalis* (De Geer) (Diptera: Muscidae) (Dethier 1955, Teskey 1969, Brodie et al. 2015).

Fly factor semiochemicals cross-attract heterospecific blow flies in the same taxonomic family (Brodie et al. 2015) but may, or may not, cross-attract—and affect oviposition—by flies in distinctively different taxonomic families, such as the calliphorid black blow flies and the muscid stable flies. Stable flies and black blow flies coinhabit livestock production facilities (Teskey 1960, Coffey 1966, Machtinger et al. 2021, 2023), with overlap in feeding and oviposition resources, such as accumulations of organic matter (e.g., manure) (Norris 1965, Stoffolano et al. 1995). Both blow flies and stable flies respond to semiochemicals emanating from conspecific excreta (Carlson et al. 2000, Brodie et al. 2015). However, it is not known whether stable flies “eavesdrop” on bacterial fly factor odors of blow flies to gauge the suitability of oviposition sites.

Using cultures of bacteria previously isolated from black blow fly excreta and shown to be attractive to blow flies (Uriel et al. 2020), we tested the hypothesis that stable flies exploit bacterial “fly factor” semiochemicals from blow flies to inform oviposition decisions.

## Materials and Methods

### Experimental Insects

Stable flies were housed in metal mesh cages (45 × 45 × 45 cm, BioQuip, Compton, CA, USA) held inside an ER-75 walk-in growth chamber (Bio Chambers Inc., Winnipeg, MB, Canada) set at 26 °C, 60% RH, and a 14:10 h (light:dark) photocycle. Flies were provisioned daily with citrated bovine blood. Gravid 7- to 10-day-old female flies were collected for bioassays approximately 2 h after they had taken a blood meal.

### Culturing of Bacteria

Bacterial isolates from black blow fly excreta (Uriel et al. 2020) were recovered from storage cultures preserved in 50% glycerol solutions held in a −80 °C freezer. Storage culture colonies were streak-plated onto plates (8.5 cm in diameter) of tryptic soy agar (TSA) (Sigma-Aldrich, St. Louis, MO, USA) to form stock plates that were stored at 4 °C. All 7 original isolates were recovered: 2 species of *Exiguobacterium* (*A* and *B*), 2 strains of *Proteus mirabilis* (*A* and *B*), 2 strains of *Morganella morganii* subsp. *sibonii* (*A* and *B*), and 1 strain of *Serratia marcescens*.

### General Bioassay Design

**Fig. 1.**
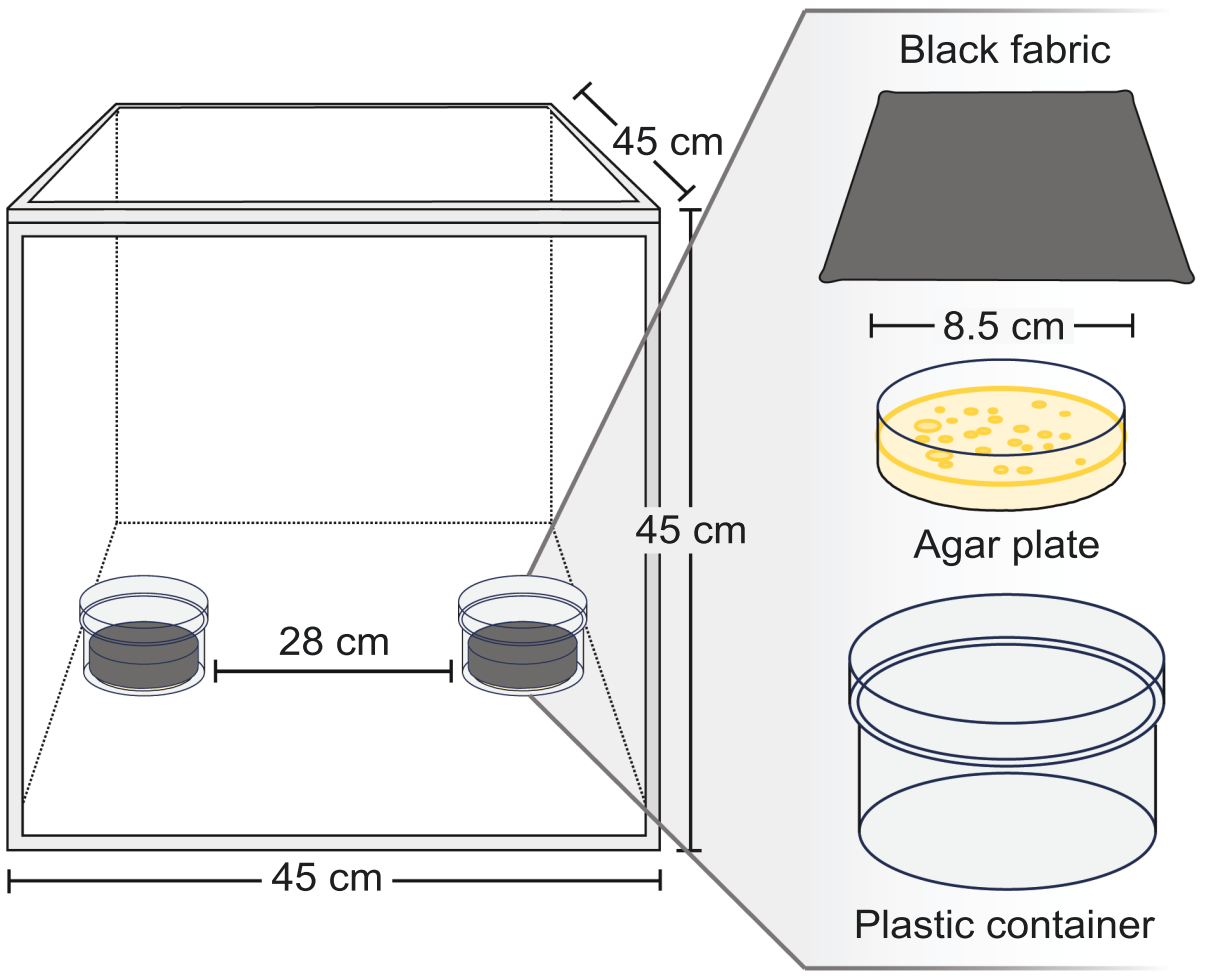
Schematic diagram (not to scale) of the 2-choice bioassay design used to test oviposition by stable flies in response to bacteria-derived odors. Stimuli consisted of sterile agar slices (control) or agar slices inoculated with 1 or 7 bacterial strains previously isolated from blow fly excreta.

In each bioassay replicate, 25 gravid female stable flies were offered a choice of paired TSA plates (8.5 cm in diameter) that had been inoculated, or not (control), with a bacterial isolate (see above) and incubated for 24 h at 30 °C. Each plate was wrapped completely with wet black cotton fabric (5 cm × 20 cm) (Fabricana, Coquitlam, BC, Canada) to present a moist and dark oviposition site (Fig. 1). Paired plates were presented in open Ziploc Twist ‘n Loc plastic containers (11.4 cm diameter, 8.3 cm height) (S. C. Johnson, Racine, WI, USA) positioned 28 cm apart on the floors of metal mesh cages (BioQuip) (Fig. 1) held in an insectary room kept at approximately 25 °C, 30–50% RH, and a 14:10 h (light:dark) photocycle. Cage floors were lined with matte brown Kraft paper (NCR Corp., Duluth, GA, USA) and illuminated with overhead grow lights (Standard Products Inc., Saint-Laurent, QC, Canada). To address potential side bias, the position of treatment and control containers was alternated between replicates.

Bioassays were terminated after 24 h by cold-euthanizing all flies and the eggs they had laid. Subsequently, the eggs from each cotton fabric square were thawed and transferred to a flat black surface to be photographed using an iPhone X (Apple, Cupertino, CA, USA). For egg counts, photographs were opened in FIJI (Schindelin et al. 2012), converted to gray scale, and made into binary (black and white) images using manually set thresholds. Binary images enhanced the contrast between eggs and their background, making it easier to identify individual “egg-objects” (groups of pixels that differed from the background and matched a predetermined size). Eggs were then counted automatically using the “analyze particles” function. For a subset of randomly selected samples, automatic egg counts were compared and confirmed by “manual” egg counts.

### Specific Experiments

To test whether the blend of all 7 bacterial isolates (7-isolate-blend) affects oviposition decision by stable flies, flies were offered a choice between a TSA plate presenting equal slices of each of the 7 bacterial isolates and a plate of sterile TSA (Exp. 1). To assess the effects of each isolate, flies were offered choices between a quarter slice of a single isolate and a quarter slice of sterile TSA (Exps. 2–8). To compare the effects of the 7-isolate-blend and the 2 strains of *M. m. sibonii*, flies were offered further choices between the 7-isolate-blend and a full plate of either *M. m. sibonii A* or *B* (Exps. 9–10).

### Statistical Analyses

Egg count data were analyzed with RStudio (Build 576 and older), using the packages *emmeans* and *lme4*, and R statistical software (v.4.1.2, R Core Team 2021). To analyze oviposition decisions by female flies, we used binomial generalized linear models, with quasibinomial errors to control for overdispersion, to compare an intercept-only model against a null model with a likelihood ratio test.

## Results

**Fig. 2.**
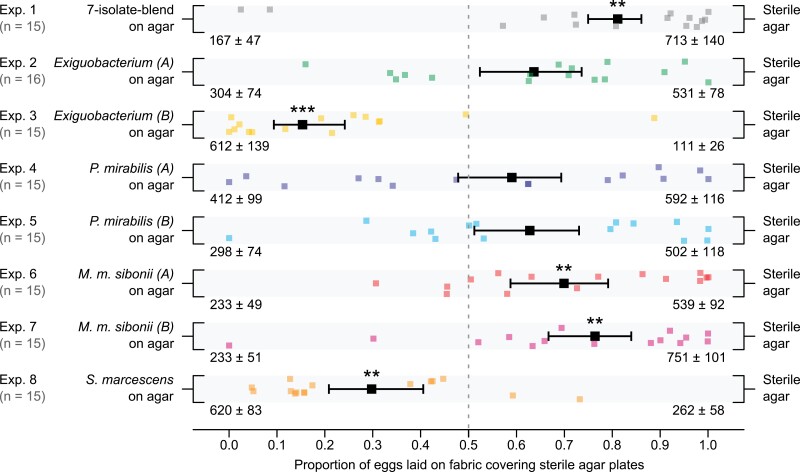
Eggs laid by stable flies on wet black fabric (Fig. 1) covering sterile agar plates or agar plates inoculated with (i) 7 bacterial strains previously isolated from blow fly excreta (7-isolate-blend; Exp. 1) or (ii) one of the 7 bacterial isolates from this blend (Exps. 2–8). In each 24-h 2-choice bioassay replicate (*n*), 25 gravid female flies were released into a bioassay cage (Fig. 1), and the eggs laid on each stimulus were counted. Small square symbols represent data of individual replicates, and large black square symbols indicate the mean proportion of eggs laid on fabric covering sterile agar plates. The mean (± SEM) number of eggs laid on each stimulus is listed on the bottom corners of the jitter plots. Asterisks (*) positioned above large black symbols denote a significant preference for a stimulus (**P < 0.01, ***P < 0.001; maximum likelihood tests).

**Fig. 3.**
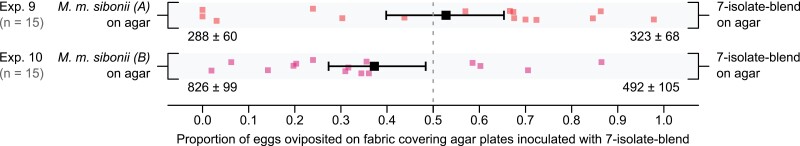
Eggs laid by stable flies on wet black fabric (Fig. 1) covering plates of agar consisting of 7 slices, each inoculated with one of 7 bacterial strains previously isolated from blow fly excreta (7-isolate-blend) or inoculated with a full agar plate of either of *M. m. sibonii A* or *B*. In each 24-h 2-choice bioassay replicate (*n*), 25 gravid female flies were released into a bioassay cage (Fig. 1), and the eggs laid on each stimulus were counted. Small square symbols represent individual replicates, and large black square symbols indicate the mean proportion of eggs oviposited on fabric covering agar plates inoculated with the 7-isolate-blend. The mean (± SEM) number of eggs laid on each stimulus is listed on the bottom corners of the jitter plots. There were no significant preferences (*P* > 0.05) for any of the test stimuli (maximum likelihood test).

Female stable flies laid significantly fewer eggs on agar presenting the 7-isolate-blend than on sterile control agar (Exp. 1: 167 ± 47 vs. 713 ± 140; *F* = 15.96, df = 1, *P* = 0.001; Fig. 2). Similarly, female flies laid significantly fewer eggs on agar inoculated with either *M. m. sibonii A* (Exp. 6) or *M. m. sibonii B* (Exp. 7) than on sterile control agar (Exp. 6: 233 ± 49 vs. 539 ± 92; *F* = 11.88, df = 1, *P* = 0.004; Exp. 7: 233 ± 51 vs. 751 ± 101; *F* = 14.29, df = 1, *P* = 0.002; Fig. 2). Conversely, female flies laid significantly more eggs on agar inoculated with *Exiguobacterium B* (Exp. 3) or *S. marcescens* (Exp. 8) than on sterile control agar (Exp. 3: 612 ± 139 vs. 111 ± 26; *F* = 32.876, df = 1, *P* < 0.001; Exp. 8: 620 ± 83 vs. 262 ± 58; *F* = 12.299, df = 1, *P* = 0.003; Fig. 2). In contrast, *Exiguobacterium A*, *P. mirabilis A*, and *P. mirabilis B* growing on agar neither induced nor deterred oviposition by female flies relative to sterile control agar (Exps. 2, 4–5, 8; all *P* > 0.05; Fig. 2). When female flies were offered oviposition choices between the 7-isolate-blend and either *M. m. sibonii A* (Exp. 7) or *M. m. sibonii B* (Exp. 8), females showed no oviposition preference in either experiment (each *P* > 0.05; Fig. 3).

## Discussion

Our data support the hypothesis that stable flies exploit “fly factor” semiochemicals from blow fly bacteria to inform oviposition decisions. Semiochemicals emitted from a blend of 7 bacterial strains isolated from blow fly excreta deterred stable fly oviposition (Exp. 1), as did the semiochemicals from each of 2 *M. m. sibonii* strains when they were tested individually (Exps. 6 and 7), with each of the 2 strains matching the effect of the 7-isolate-blend (Exps. 9 and 10).

As the 2 strains of *M. m. sibonii*, which deterred oviposition by stable flies (Exps. 6 and 7), were attractive to blow flies (Uriel et al. 2020), it is conceivable that the deterrent response by stable flies may help reduce interspecific competition. This interpretation is supported by findings that *Exiguobacterium B* was one of 2 isolates from blow fly feces that failed to attract blow flies (Uriel et al. 2020) but induced oviposition by stable flies (Exp. 3).

Oviposition deterrence caused by the presence of con- and heterospecifics in otherwise suitable sites for larval development is well documented. Various dipterans, including stable flies, avoid oviposition on substrates that bear chemical and bacterial cues indicative of prior colonization by flies (Lam et al. 2007, Nayduch 2017, Baleba et al. 2019). Inoculation of prospective stable fly oviposition sites with blow fly bacterial symbionts may distort the semiochemical profile typically associated with stable fly larval habitats. This prediction could be tested by analyzing, and behaviorally testing, headspace volatiles of potential oviposition sites before and after bacterial inoculation (Logan and Birkett 2007).

Preferential oviposition by female stable flies on sites inoculated with *S. marcescens* is difficult to interpret. Although *S. marcesens* has previously been isolated from natural habitats of stable fly larvae (e.g., hay and horse manure mixtures), *S. marcesens* alone failed to elicit oviposition by female stable flies and sustain growth of stable fly larvae (Romero et al. 2006). Furthermore, *S. marcescens* is deemed a facultative pathogen to stable flies, expressing lethal effects on early larval instars (Watson and Petersen 1991, Lysyk et al. 2002). The contrasting results obtained by Romero et al. (2006) and in our study (Exp. 8) may be explained by (i) potential differences in metabolites produced by bacteria growing on TSA (our study) and phosphate-buffered saline or natural substrates (Romero et al. 2006), and (ii) differences in bacterial densities that were tested. For example, production of toxic enzymes by *Serratia* spp. appears dependent on the culture media on which bacteria are grown (Lysyk et al. 2002). Furthermore, *Klebsiella oxytoca* bacteria—deposited by female house flies on their eggs and proliferating over time—density-dependently induce or deter further oviposition by other female house flies (Lam et al. 2007).

There is an ever-growing body of literature reporting associations between stable flies and their bacterial symbionts. Bacteria-mediated effects on stable fly foraging and oviposition behaviors include (i) attraction of foraging flies to bacteria in the bovine skin microbiome (Nayani et al. 2023b), (ii) increased oviposition by flies on substrates inoculated with bacteria isolated from natural larval habitat (i.e., bale feeder residue) (Romero et al. 2006), and (iii) fecal bacterial loads being positively correlated with oviposition site selection by female flies and larval development (Talley et al. 2009, Albuquerque and Zurek 2014, Friesen et al. 2016).

The semiochemicals emitted by bacterial strains that induced or deterred oviposition by stable flies in our study could be identified and developed as stable fly control tactics. Attractive semiochemicals could be formulated as a lure to enhance captures of flies in vision-based traps (Zhu et al. 2016). Conversely, proper formulations of deterrent bacterial semiochemicals could be utilized to render otherwise suitable oviposition sites unacceptable or to reduce the attractiveness of hosts (Lucas-Barbosa et al. 2022). Concurrent deployment of both tactics may enhance the efficacy of a push–pull strategy (Cook et al. 2007, Logan and Birkett 2007) for stable fly control.
